# Regional Spatial Management Based on Supply–Demand Risk of Ecosystem Services—A Case Study of the Fenghe River Watershed

**DOI:** 10.3390/ijerph17114112

**Published:** 2020-06-09

**Authors:** Hongjuan Zhang, Juan Feng, Zhicheng Zhang, Kang Liu, Xin Gao, Zidong Wang

**Affiliations:** 1 College of Urban and Environmental Sciences, Northwest University, Xi’an 710127, China; 201710179@stumail.nwu.edu.cn (H.Z.); 201820796@stumail.nwu.edu.cn (J.F.); zhangzhicheng1@stumail.nwu.edu.cn (Z.Z.); 2 Shaanxi Key Laboratory of Earth Surface System and Environmental Carrying Capacity, Xi’an 710127, China; 3 Business School, Hohai University, Nanjing 211100, China; gxtz1987@hhu.edu.cn; 4 School of Economics and Management, China Three Gorges University, Yichang 443000, China; wzd0126@163.com

**Keywords:** food provision, water yield, soil retention, climate regulation, supply–demand ratio, risk zoning

## Abstract

The supply–demand risk assessment of ecosystem services (ES) can identify the supply–demand risk level, which is very important for the sustainable management of regional ES. In this study, taking the Fenghe River watershed (FRW) as a case, based on the status and the change trend of the supply–demand ratio of ES, and the ES supply change trend, the supply–demand risk level of food provision (FP), water yield (WY), soil retention (SR), and climate regulation (CR) are evaluated, and the risk management zones of the FRW are divided using spatial superposition. The results show that: (1) The supply and demand of SR are spatially matched, while the other three ES are spatially mismatched. (2) From 2000 to 2015, the supply amount of FP, WY, and SR increases by 11.59%, 1.25% and 55%, respectively, while the supply amount of CR decreases by 5.15%. At the same time, the demand amount of FP, WY, SR and CR increases by 39.97%, 53.88%, 36.3% and 215.5%, respectively. (3) The supply–demand ratio means of four ES in the FRW are all greater than 0, but there are some areas within that are less than 0. (4) In terms of sub-watershed scale, except for SR, there are critically endangered areas for the other three ES. Moreover, the FRW is divided into 11 supply–demand risk management zones, such as FS-WY-CR critically endangered zone, WY-CR critically endangered and FS vulnerable zone. The supply–demand risk management zones based on multiple ES can identify the risk level of each ES in each zone. These results and conclusions can provide the basis for rational allocation of resources and sustainable management of ES.

## 1. Introduction

Ecosystem services (ES) refer to the ecological characteristics, functions, or processes that directly or indirectly contribute to human wellbeing [1,2,3]. The concept of ES links ecological and socioeconomic systems [4,5]. Further, ES are affected by the ecosystem properties, and at the same time, they are also affected by socioeconomic development [2,6]. With the rapid growth of population and the development of social economy, humans’ demands for ES increase, and ES are in short supply [7,8,9]. At the same time, the excessive use of ES by mankind poses a threat to the sustainable development of the ecosystem and human society [10]. More than 60% of global ES have shown a downward trend, and this trend is not likely to be suppressed in the next 50 years [11]. Some studies have shown that the imbalance of ES supply–demand is the potential reason for ecosystem degradation [12,13]. Therefore, identifying the supply–demand matching and the supply–demand risk level of ES can provide intuitive and effective information for regional environment management and decision making [14,15].

The quantitative assessment of ES supply and demand is the key to identify the supply–demand risk of ES. The ES supply refers to the capacity to provide ecosystem goods and services within a given time period and a particular area [4]. To date, a large quantity of literature has evaluated global or regional ES supply in different methods [13,15]. The evaluation of ES supply mainly adopts the market-valuing method [15,16] or the model simulation method [17,18]; in particular, the InVEST (Integrated Valuation of Ecosystem Services and Tradeoffs) model that evaluates ES supply in spatial explicit form has been widely used [19,20]. These evaluations provide a basis for studying the supply–demand relationship of ES. Compared with ES supply, the research of ES demand has received significant attention in recent years [16]. Within the reviewed studies, the concept of ES demand is defined from two different perspectives [4,20,21,22]. The first perspective is that ES demand is the actual use or consumption of a good or service [4]. The second perspective denotes that it is the level required or desired by individual or society preferences for ES specific attributes [21,22]. From the perspective of consumption, provisioning services demand is the amount of ES (e.g., food, fresh water) consumed or used in a specific time and space [4]. From the perspective of social preferences, regulating service demand is defined as the quantity or quality of ES (e.g., water quality, air quality) that are required or desired by society [23], and the cultural service demand is expressed as individuals’ preference or expectation (e.g., most important perceived ES, willingness to pay) for some service attribute [22,23,24]. For example, Palomo et al. use the most important perceived ES to assess 25 ES demand through two expert workshops [23]. Nahuelhual et al. apply willingness to pay to estimate agricultural heritage values [24]. Based on these definitions, various assessment methods for ES demand have been explored, such as the consumption method [25], the preference method [26,27,28], the contingent valuation method [29], and the matrix method [4,12]. Although these methods have promoted the progress of ES demand assessment, some obstacles still exist. At present, there is still a lack of fully quantitative assessment method for ES demand. Based on the descriptions of ES supply and demand assessment methods, there are differences between supply and demand assessment methods. Sometimes, there is inconsistency between the assessment units of supply and demand.

Recently, the research of risk assessment based on ES has attracted extensive attention from researchers, and it mainly includes two aspects. One of them is the risk assessment based on ES supply [30,31,32]. For example, Dong et al. [32] divided risks into five levels: no risk, mild, moderate, high, and extremely high risk based on regional ecological risk early warning frameworks of the ES supply. The other aspect is the risk assessment based on the supply–demand relationship of ES [33,34]. Maron et al. [34] proposed a conceptual framework of ES threat, which considered the current status and change trend of ES supply–demand ratio and the change trend of ES supply. This conceptual framework divides the ES threat into four levels, which are “secure”, “at risk”,” undersupplied”, and “lost”, and then refines these four levels into seven small levels. The advantage of this risk assessment method is that it not only considers the current status of supply and demand of ES, but also considers their change trend. However, due to the limitation of the difficulty of ES demand assessment, the supply–demand risk case studies are relatively lacking. Further, the study of regional management based on the division of supply–demand risk of multiple ES needs to be explored.

To supplement the lack of studies on the supply–demand risk of multiple ES, based on the supply–demand risk of various ES, the risk management zones of Fenghe River watershed (FRW) is carried out. According to the research on the importance of ES in the early stages of FRW [35], and considering the limitations of the data obtained, this study selects food provision (FP), water yield (WY), soil retention (SR), and climate regulation (CR) to assess their supply–demand risks. The three objectives of this study are as follows: (1) to identify the spatiotemporal distributions and changes of four ES supply and demand; (2) to identify the supply–demand ratios and their change trends of four ES; (3) to identify the supply–demand risk of four ES and divide risk management zones based on supply–demand risk of four ES. Finally, this study discusses the causes of supply–demand risk of ES and the applications of supply–demand risk management zones.

## 2. Materials and Methods

### 2.1. Study Area

**Figure 1:**
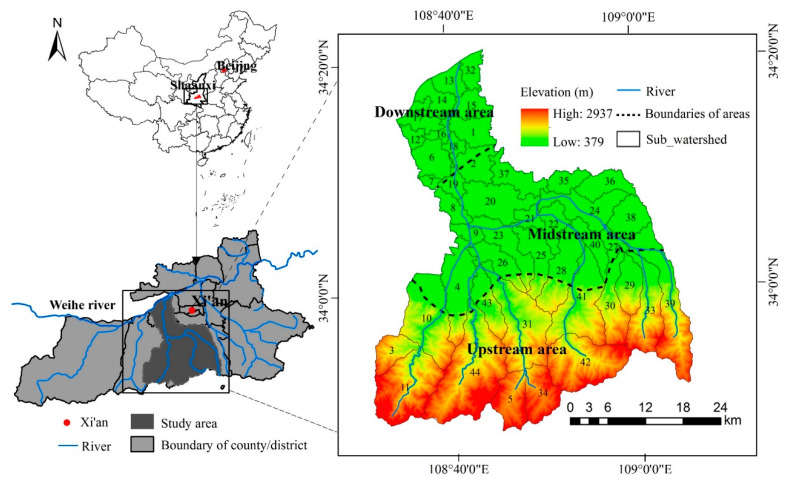
The location of the Fenghe River watershed (FRW).

FRW, located in Xi’an, Shaanxi Province, China (Figure 1), has a temperate monsoon climate. It has an average annual temperature of about 15 °C and an average annual rainfall of about 800 mm, and the precipitation is concentrated from July to October. From the landform type, the north side of the FRW belongs to the Weihe Plain and the south side of that belongs to the Qinling Mountain. The FRW is close to the main urban area of Xi’an City. The nearest place is about 3 km away from the center of Xi’an City, and the farthest place is about 55 km away from the center of Xi’an city. The administrative center of Chang’an District of Xi’an is also located in this watershed [35].

**Figure 2:**
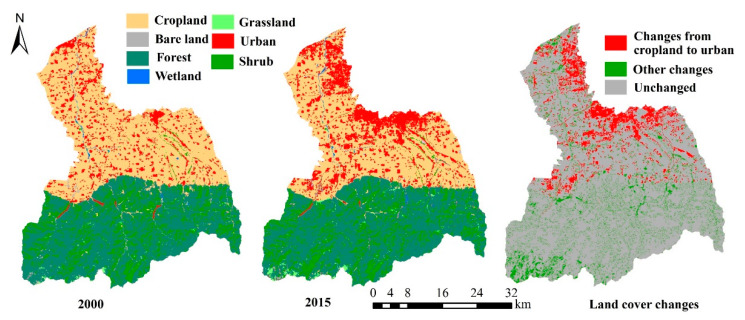
Spatial distributions and changes of land cover in the FRW from 2000 and 2015.

The permanent population of the FRW was about 880,000 in 2000, and about 1,200,000 in 2015, which is an increase of about 40%. The population density increased from about 500 persons/km^2^ in 2000 to 700 persons/km^2^ in 2015. From 2000 to 2015, GDP increased by 5.13 million CNY (from 480,100 CNY to 5,613,100 CNY). The changes of land cover/use type in the FRW are mainly caused by urbanization. The urban area changed from about 126 km^2^ in 2000 to 241 km^2^ in 2015, showing a growth rate of 90%. According to Figure 2, the land use types of the FRW are mainly forest, shrub and cropland, and it also can be seen that the cropland in the midstream area and downstream area is transformed into urban land by comparing the land use types in 2000 and 2015. In the context of urbanization, studies on whether the supply and demand of ES have changed and whether they match will play a reference role in formulating watershed management policies.

### 2.2. Data Sources

In this study, four key ES are selected, including FP, WY, SR, and CR. The data of land use, precipitation, potential evapotranspiration, soil properties, food production, carbon storage, DEM and NDVI (Normalized Difference Vegetation Index) are used to evaluate four ES supply. The population data, nighttime lights, food calories per capita, per capita carbon emissions, and water consumption data are used to assess four ES demand. The per capita carbon emission needs to be calculated by the multi energy consumption data. The sources of these data are listed in Table 1.

### 2.3. Mapping ES Supply and Demand

#### 2.3.1. Population Spatialization

Based on nighttime lights and land use, the population is spatialized in this study [41,42,43]. In order to make each grid contain only one type of land use as much as possible, this study established a 50 m × 50 m fishing net to cover all the townships involved in the FRW. Then, we use the grid data processing method of Guo et al. [42] to obtain the 50 m × 50 m grid population at the township scale. The specific processing steps are as follows.

Firstly, basic data acquisition. The township population data *P_i_* of each 50 m × 50 m grid, and the nighttime light value *L_i_* and building area data *B_i_* corresponding to the center point are extracted by using the center point of the fishing net.

Secondly, calculating the weight of each grid population. The nighttime light data is regarded as the height of the building, and the weight *W_i_* of a single grid is equal to the building area *B_i_* of the grid multiplied by the corresponding nighttime light data *L_i_* (Equation (1)). Then, taking the township as the unit, the grid weights *W_i_* and ∑*W_i_* included in each township are obtained. The actual weight of each grid *W_i_*′ is equal to *W_i_* divided by ∑*W_i_* (Equation (2)).

(1) $$W_{i}=B_{i}\times L_{i}$$

(2) $$W_{i}^{\prime }=W_{i}/\sum W_{i}$$

Finally, the population of each 50 m × 50 m grid *P_i_*′ is equal to the actual weight of the grid *W_i_*′ multiplied by the actual population of the corresponding township *P_i_* (Equation (3)).

(3) $$P_{i}^{\prime }=P_{i}\times W_{i}{}^{\prime }$$

#### 2.3.2. Food Provision

##### Supply

To calculate the supply of various types of crop products (e.g., rice, meat, wheat, apple, grape) consistently, we applied the food provision calories method to convert crop yield into heat value [44]. The food provision can be mapped using the Equation (4) at a 50 m × 50 m resolution [12].

(4) $$E_{s}=\sum_{i=1}^{n}E_{i}=\sum_{i=1}^{n}\left(10\times Y_{i}\times A_{i}\times Cal_{i}\right)$$

where *E_s_* is the total calories of the FP (kcal); *i* is the number of crop types from 1 to *n*; *E_i_* is the heat value of the *i-th* crop type; *Y_i_* (kg/hm^2^) is the yield per unit area of *i* crop; *A_i_* is the area of *i* crop; and *Cal_i_* is the calories per 100g of *i* crop.

##### Demand

Human beings are the main objects of food consumption. We use the minimum energy needed for normal survival to quantify the demand for FP. The food demand is can be calculated using the following equation.

(5) $$E_{d}=\sum_{i=1}^{n}POP_{i}\times Cal_{p}\times 365$$

where *E_d_* is the minimum living energy required by all people in the FRW; *i* refers to the *i-th* grid; *POP_i_* is the population of grid *i*; *Cal_p_* is the minimum energy per person per day. Based on the “China food and Nutrition Development Program (2001–2010)”and “China food and Nutrition Development Program (2014-2020)”, the daily consumption per capita is 2300 kcal.

#### 2.3.3. Water Yield

##### Supply

We used the InVEST model to evaluate WY. The WY evaluation of InVEST model is based on the water balance equation. The annual water output of each grid unit is the precipitation minus the actual evapotranspiration [45]. We mapped annual WY (*Y_x_*) in each 50 m × 50 m grid cell on pixel *x* as follows.

(6) $$Y_{x}=\left(1-\frac{AET_{x}}{P_{x}}\right)\times P_{x}$$

where *Y_x_* is the annual WY for pixel *x*; *AET_x_* is the annual actual evapotranspiration in pixel *x*; and *P_x_* is the annual average precipitation for pixel *x*.

##### Demand

Fresh water demand includes humans’ freshwater demand and river ecological flow requirements in a specific area. The humans’ freshwater demand is divided into domestic consumption, agricultural consumption, industrial consumption, urban public consumption, and ecological consumption. The humans’ water demand is spatialized based on the type of land use and population. The river ecological flow requirements refer to the average flow of the river to maintain good ecological function, usually accounting for 30% of the average annual discharge [46]. We used formula (7) to calculate the fresh water demand.

(7) $$FW_{d}=Q_{p}+Q_{a}+Q_{i}+Q_{u}+Q_{e}+F\times 30\%$$

where *FW_d_* is the total fresh water demand; *Q_p_* is the domestic consumption; *Q_a_* is the agricultural consumption; *Q_i_* is the industrial consumption; *Q_u_* is urban public consumption, including water demand for construction and service industries; *Q_e_* is ecological consumption, such as water for dust control in cities; and *F* is the annual discharge (m^3^).

#### 2.3.4. Soil Retention

Soil retention supply refers to the erosion control ability of ecosystems (e.g., forest, paddy field, grassland) to prevent soil loss through its structure and process [47]. Based on the revised universal soil loss equation (RUSLE) [48,49], the SR supply is obtained by the difference between the potential soil erosion amount and the actual soil erosion amount. The actual amount of soil erosion is the amount of soil erosion that humans expect to be managed, so the actual amount of soil erosion is used as the demand for SR [50]. The calculation equation is as follows.

(8) $$RKLS_{x}=R_{x}\cdot K_{x}\cdot LS_{x}$$

(9) $$USLE_{x}=R_{x}\cdot K_{x}\cdot LS_{x}\cdot C_{x}\cdot P_{x}$$

(10) $$SEDRET_{x}=RKLS_{x}-USLE_{x}$$

where *SEDRET_x_* is the amount of SR supply of grid *x*; *RKLS_x_* is the potential soil erosion amount of grid *x*; *USLE_x_* is the actual soil erosion amount of grid *x*; *R_x_* is the rainfall erosivity factor; *K_x_* is the soil erodibility factor; *LS_x_* is the slope length factor; *C_x_* is the vegetation coverage factor; *P_x_* is the soil and water conservation measure factor. *USLE_x_* is also the demand for SR.

#### 2.3.5. Climate Regulation

##### Supply

The supply of CR is represented by carbon storage. We used the InVEST model to calculate carbon storage. It consists of four parts: aboveground carbon storage, underground carbon storage, soil organic carbon storage and dead organic carbon storage. Carbon storage data are obtained by consulting the existing literature [51]. The Equation (11) is used to calculate the supply of carbon storage.

(11) $$C_{s}=C_{above}+C_{below}+C_{soil}+C_{dead}$$

where *C_s_* is the total carbon storage (t/hm^2^); *C_above_* is the aboveground biological carbon storage; *C_below_* is the underground biological carbon storage; *C_soil_* is the soil carbon storage; *C_dead_* is the dead organic carbon storage.

##### Demand

The demand for CR is measured by carbon emissions. Carbon emissions are calculated based on per capita carbon emissions and gridded population. The carbon emission of Xi’an is calculated by multiplying each energy consumption by its carbon emission coefficient. The per capita carbon emission of Xi’an is obtained by dividing the carbon emissions of Xi’an by the resident population of Xi’an (Equation (12)). Finally, based on the gridded population, the carbon dioxide demand data of the FRW is obtained, and then the carbon demand is obtained by multiplying 0.2727 coefficient (Equation (13)).

(12) $$D_{cp}=\left(\sum_{i=1}^{n}C_{i}\times EF_{i}\right)/POP_{t}$$

(13) $$D_{c}=D_{cp}\times \rho_{pop}\times 0.2727$$

where *D_cp_* is the per capita carbon dioxide emission; *C_i_* is the consumption of *i-th* type energy; *n* is the number of energy types consumed; *EF_i_* is the carbon emission factor of the *i-th* energy; *POP_t_* is the permanent resident population of Xi’an in *t* period; *D_c_* is the demand for carbon emissions; *ρ_pop_* is the gridded population. Finally, the per capita carbon emission is about 1.12 t/person in 2000 and 2.53 t/person in 2015.

### 2.4. Supply and Demand Relationship

There are three kinds of relationship between ES supply and ES demand: deficit, surplus and balance. We applied the ES supply–demand ratio (ESDR) indicator to calculate the relationship between ES supply and ES demand through Equation (14) [52,53].

(14) $$ESDR=\frac{ESS-ESD}{\left(ESS_{max}+ESD_{max}\right)/2}\{\begin{array}{c} >0,\;surplus\\ =0,\;balance\\ <0,\;deficit \end{array}$$

where *ESDR* is the ES supply–demand ratio; *ESS* refers to actual supply for a specific ES; *ESD* refers to human demand for a specific ES; *ESS_max_* and *ESD_max_* refer to the maximum value of actual supply and human demand for a specific ES, respectively. A positive ESDR value shows supply surplus, a value of zero shows the supply–demand balance, and a negative value shows supply deficit.

### 2.5. The Supply–Demand Risk of ES

The ES threat assessment framework proposed by Maron et al. [34] integrates the current status and the change trend of ESDR, and the change trend of ES supply to assess the supply–demand risk of ES. In this study, according to the natural ecological environment of the FRW and the ES threat assessment framework proposed by Maron et al. [34], the loss and failure risk is removed, and the supply–demand risk of ES is divided into five levels (Table 2). The levels from high to low are critically endangered, endangered, stable but undersupplied, vulnerable, and secure, respectively. Among them, critically endangered level, endangered level and stable undersupplied level belong to the supply deficit. The vulnerable level and secure level belong to supply surplus. At the same time, the critically endangered level, endangered level, and stable but undersupplied level are also called high risk level, and the vulnerable level and secure level are also called low risk level. Finally, taking sub-watershed as the scale of spatial management, the supply–demand risks of four ES are superposed, and the supply–demand management zones of the FRW are obtained.

## 3. Results

### 3.1. Spatiotemporal Distribution and Changes of ES Supply

#### 3.1.1. Food Provision

**Figure 3:**
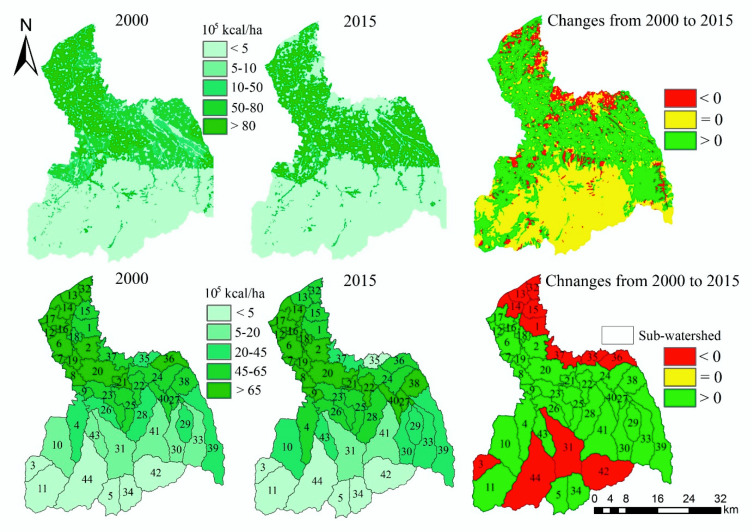
The spatial distribution and spatiotemporal changes of food provision (FP).

In general, the spatial distribution and spatiotemporal change trend of grid scale and sub-watershed scale are almost consistent (Figure 3). The high-value areas of FP are mainly distributed in the cropland areas of the midstream area and downstream area. The amount of FP in 2015 (2.41 × 10^12^ kcal) increased by 11.59% compared with 2000 (2.16 × 10^12^ kcal) (Table A1). From 2000 to 2015, there are obvious reduction areas in the midstream area and downstream area. However, in the middle and downstream regions, FP also has increased areas.

In terms of sub-watershed scale (Figure 3), the amount of FP in the sub-watersheds of the midstream area and downstream area is higher. In contrast, the amount of FP in the sub-watersheds of the upstream area is lower. From 2000 to 2015, it can be identified that the FP capacity of sub-watersheds close to the main urban area of Xi’an has declined significantly.

#### 3.1.2. Water Yield

**Figure 4:**
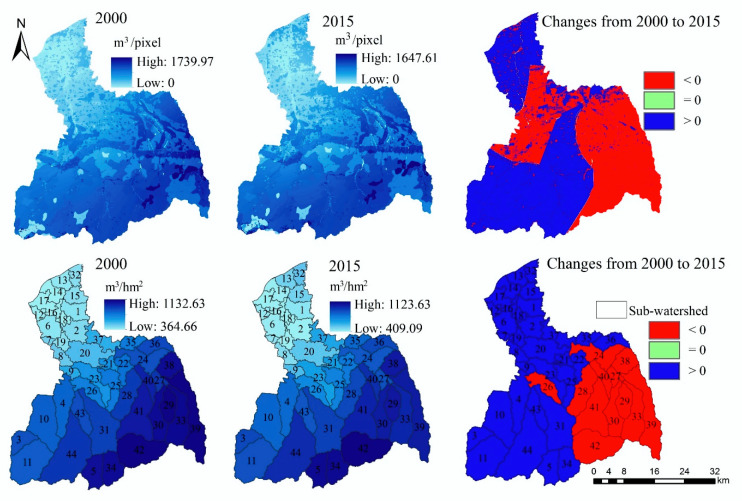
The spatial distribution and spatiotemporal changes of WY.

The high-value areas of WY in the FRW are mainly distributed in the southeast, while the low-value areas are mainly distributed in the northwest (Figure 4). Compared with 2000, the amount of WY increased by 1.2% in 2015 (from 5.86 × 109 m^3^ to 5.93 × 109 m^3^) (Table A1), and it decreased in the southeast of the upstream area and increased in the southwest. In addition, the central region of the watershed also presents a large area of reduction.

From the sub-watershed scale (Figure 4), the WY ability of the southeast sub-watershed is higher, and it gradually decreases from southeast to northwest. Compared with 2000, the amount of WY in the southeast sub- watersheds decreased in 2015, while that of other sub-watersheds increased.

#### 3.1.3. Soil Retention

**Figure 5:**
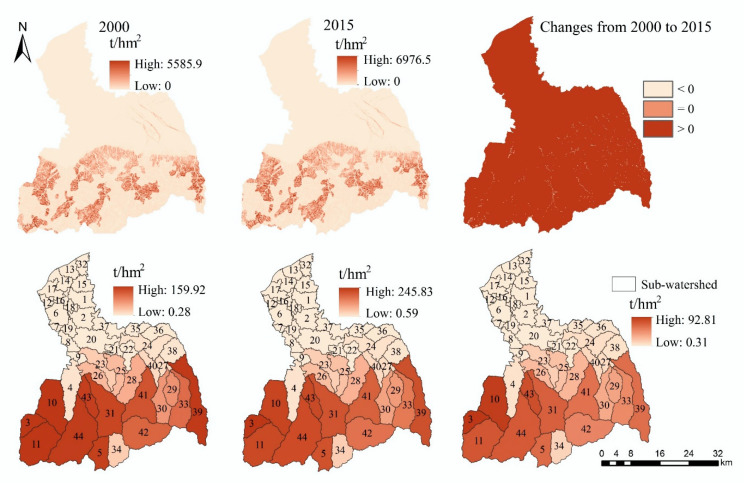
The spatial distribution and spatiotemporal changes of soil retention.

In 2000 and 2015, the high-value areas of SR are all distributed in the southern mountainous areas, and the spatial distribution is almost the same (Figure 5). From 2000 to 2015, the amount of SR increased by 55% (from 1.33 × 10^8^ t to 2.07 × 10^8^ t) (Table A1), and it has increased in most areas of the FRW, but only decreased in a small number of areas.

From the sub-watershed scale (Figure 5), the amount of SR decreases gradually from south to north, and the amount of SR in the plain area is very small. From 2000 to 2015, the increased sub-watersheds of SR are mainly distributed in the upstream area of the FRW.

#### 3.1.4. Climate Regulation

**Figure 6:**
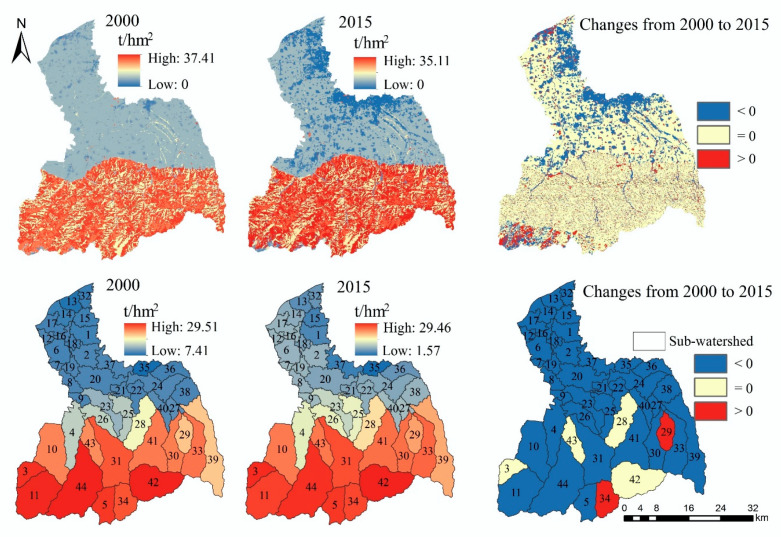
The spatial distribution and spatiotemporal changes of climate regulation (CR).

In 2000 and 2015, the high-value areas of carbon storage are distributed in the upstream area, while the low-value areas of carbon storage are mainly distributed in the midstream area and downstream area (Figure 6). From 2000 to 2015, the areas with reduced carbon storage are mainly distributed in the midstream area and downstream area. The carbon storage decreased from 1.32 × 10^7^ t in 2000 to 1.25 × 10^7^ t in 2015, a decrease of 5.15% (Table A1).

From the perspective of the sub-watershed, the amount of carbon storage gradually decreases from south to north. The amount of carbon storage has not change significantly in the 3, 28, 42 and 43 sub-watersheds, only increasing in the 29 and 34 sub-watersheds and decreasing in other sub-watersheds.

### 3.2. Spatiotemporal Distribution and Changes of ES Demand

#### 3.2.1. Spatialization of Population

**Figure 7:**
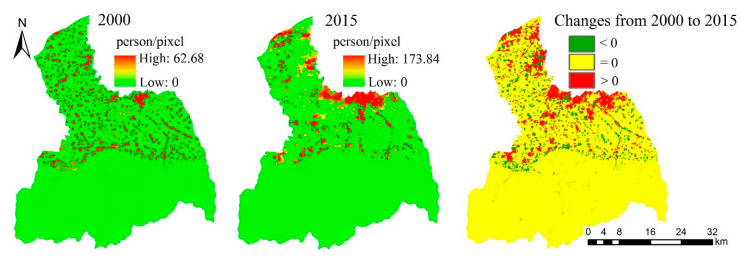
The spatial distribution and change of population in FRW in 2000 and 2015.

The population of the FRW is about 886,800 in 2000 and 1,235,900 in 2015, an increase of 39.37%, and the increased areas are mainly located in the middle and downstream areas of the FRW, especially the areas close to the main urban area of Xi’an City (Figure 7).

#### 3.2.2. Food Demand

**Figure 8:**
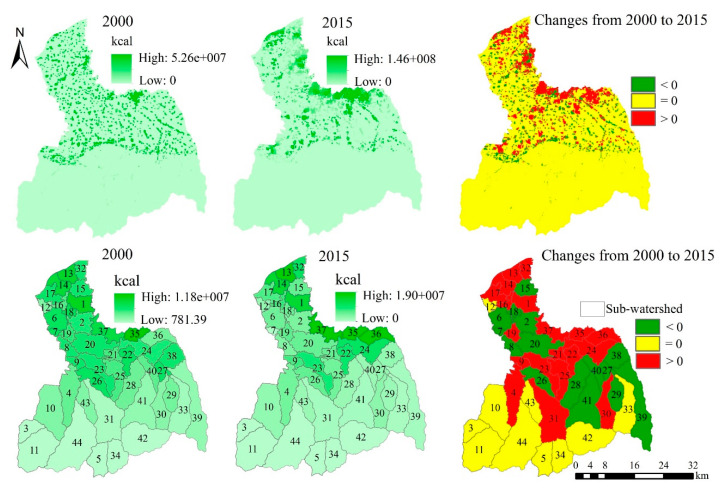
The spatial distribution and spatiotemporal changes of food demand.

From the grid scale, the high-value areas of food demand are mainly distributed in the midstream area and downstream area (Figure 8). Compared with 2000, the high-value areas of food demand in 2015 have increased, and the main increase areas are distributed close to the main urban area of Xi’an. From 2000 to 2015, the amount of food demand increased from 7.45 × 10^11^ kcal to 10.38 × 10^11^ kcal, an increase of 39.37% (Table A2). From the sub-watershed scale, the amount of food demand in sub-watersheds 1, 13, 14, 35, and 37 are higher, while it is lower in sub-watersheds 3, 5, 11, 34, et al. From 2000 to 2015, there is a significant increase for food demand in the sub-watersheds 13, 35, 36, and 37, while there is a significantly decrease in the sub-watersheds 7, 27, and 38.

#### 3.2.3. Fresh Water Demand

**Figure 9:**
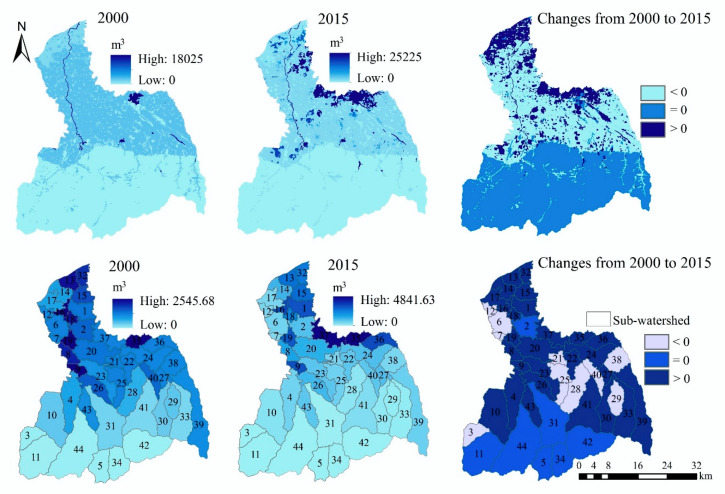
The spatial distribution and spatiotemporal changes of fresh water demand.

From the grid scale, the high-value areas of fresh water demand are mainly distributed in urban areas and river areas, followed by cropland areas, while the low-value areas are mainly distributed in the southern mountains (Figure 9). Compared with 2000, the increased areas are mainly distributed in the midstream area and downstream area in 2015, and the fresh water demand has increased by 53.88% (from 2.45 × 10^8^ m^3^ to 3.77 × 10^8^ m^3^) (Table A2). From the perspective of the sub-watershed scale, the high-value areas of fresh water demand are distributed in sub-watersheds 8, 9, 13, 16, 18, 19, and 35 in 2000, while they are distributed in the sub-watersheds 1, 15, 32, 36, and 37 in 2015. The fresh water demand is lower in the sub-watersheds of upstream area in 2000 and 2015, such as sub-watersheds 3, 5, 11, 34, 42, and 44.

#### 3.2.4. Soil Erosion

**Figure 10:**
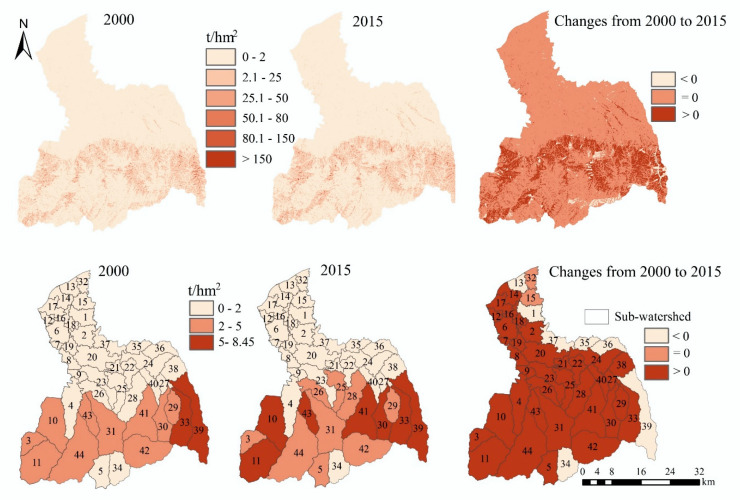
The spatial distribution and spatiotemporal changes of carbon emissions.

The high-value areas of soil erosion are distributed in the upstream area (Figure 10). From the perspective of the soil erosion modulus, most areas of the FRW are slightly eroded, and there is some mild to moderate soil erosion in the upstream area. From 2000 to 2015, the amount of soil erosion in most areas has not changed, and its increased areas are mainly located in the upstream area, with a total increase of 36.3% (from 4.38 × 10^6^ t to 5.97 × 10^6^ t) (Table A2). From the perspective of sub-watershed scale, in 2000, only the sub-watersheds 33 and 39 were more than 5 t/hm^2^, while in 2015, the sub-watersheds 10, 11, 30, 33, 39, 41, and 43 were all more than 5 t/hm^2^. From 2000 to 2015, the amount of soil erosion in the sub-watersheds 15 and 32 do not change significantly, and it decreases in the watersheds 1, 13, 34, 35, 36, 37, and 39, while it increases in the other sub-watersheds.

#### 3.2.5. Carbon Emission

**Figure 11:**
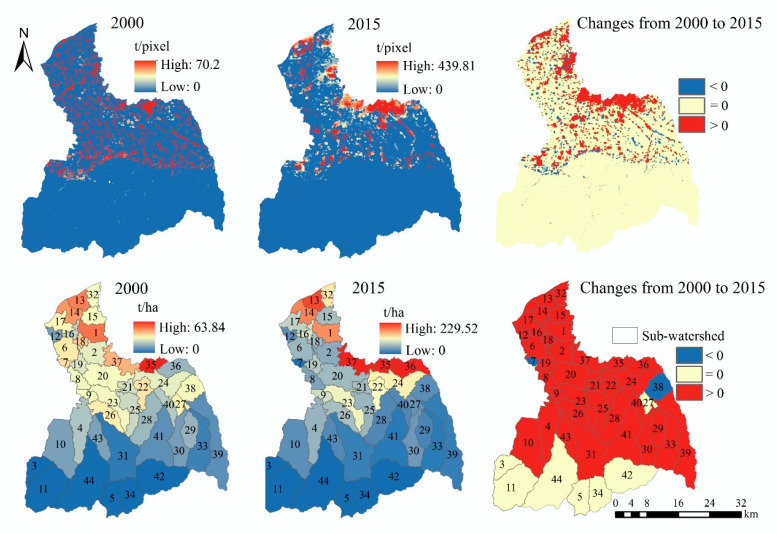
The spatial distribution and spatiotemporal changes of carbon emissions.

The high-value areas of carbon emissions are mainly located in the urban areas in the midstream area and downstream area of the FRW (Figure 11). From 2000 to 2015, the amount of carbon emissions increases significantly in the areas close to the main urban area of Xi’an. The amount of carbon emissions increased from 9.93 × 10^5^ t in 2000 to 3.13 × 10^6^ t in 2015, an increase of 2.14 × 10^6^ t and 215.51% (Table A2). From the sub-watershed scale, only the sub-watershed 35 has higher carbon emissions in 2000, while in 2015, the sub-watersheds 13, 35, 36, and 37 all have higher carbon emissions.

### 3.3. The Supply–Demand Matching of ES

#### 3.3.1. The Supply–Demand Matching of Grid Scale

**Figure 12:**
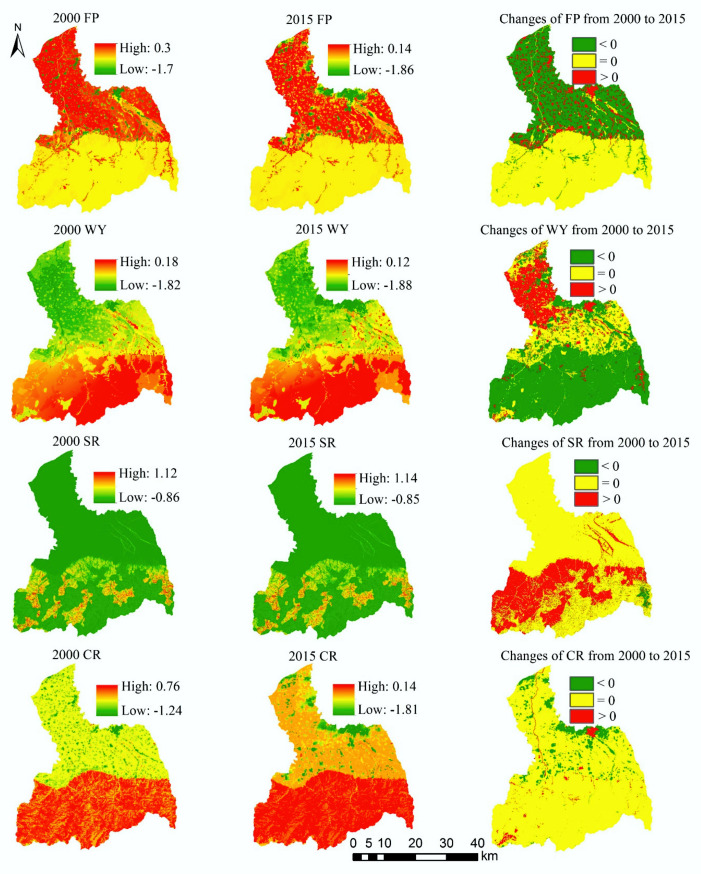
The spatial distribution and spatiotemporal change of the supply–demand ratio in grid-scale.

There are spatiotemporal differences in the supply–demand ratio for four ES (Figure 12). The high-value areas of the supply–demand ratio for FP are mainly distributed in the midstream area and downstream area. Compared with 2000, the supply–demand ratio of FP in 2015 is almost unchanged in the upstream area, while the increase area and decrease area of the supply–demand ratio for FP are distributed in the midstream area and downstream area. Among them, the supply–demand ratio of FP on cropland shows an increasing trend. The high-value areas of the supply–demand ratio for WY are mainly located in the upstream area, and the supply–demand ratio of WY in the midstream area and downstream area is relatively low. Compared with 2000, WY’s supply–demand ratio declines in the upstream area in 2015, remains unchanged in the midstream area, and increases in the downstream area.

The high-value areas of the supply–demand ratio for SR are mainly located in the upstream area. From 2000 to 2015, the supply–demand ratio of SR in the midstream area and downstream area remains almost unchanged, while the supply–demand ratio of SR appears as rising in the southwest of the upstream area. The supply–demand ratio of CR is greater than 0 in the upstream area, that is, supply surplus. The supply–demand ratio of CR is relatively low in the midstream area and downstream area, but the supply–demand ratio in most areas is still greater than 0. From 2000 to 2015, the supply–demand ratio of CR remains unchanged in most regions, but decreases in the urban areas.

From the overall perspective of the FRW, the supply–demand ratio mean of the four ES is greater than 0 (Table 3), that is, supply surplus. However, except for a slight increase in the supply–demand ratio of SR, the supply–demand ratios of other three ES show a downward trend.

#### 3.3.2. The Supply–Demand Matching of Sub-Watershed Scale

**Figure 13:**
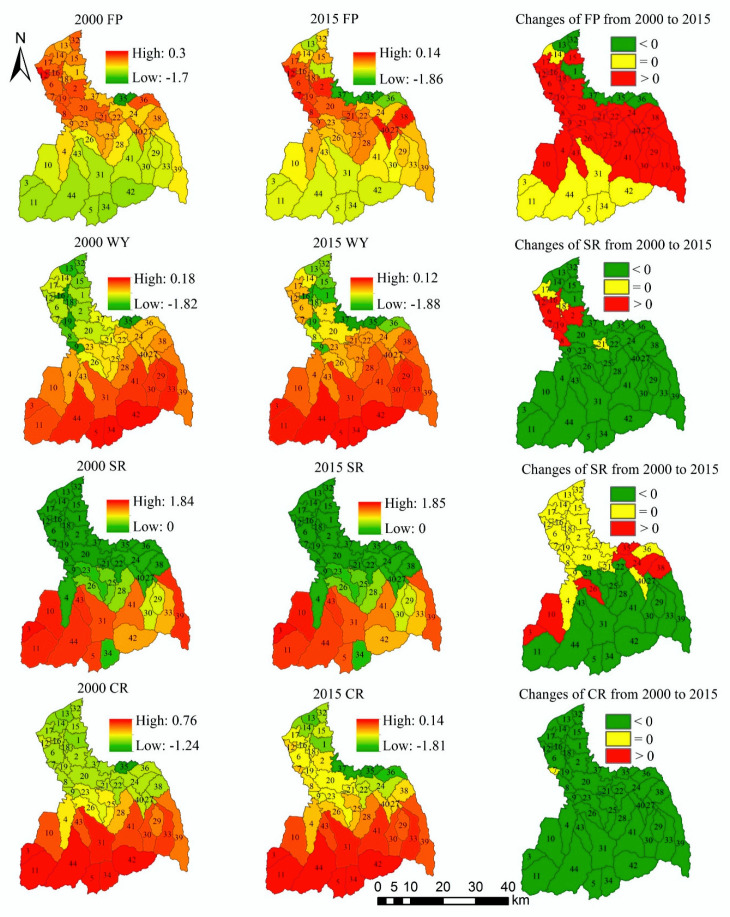
The spatial distribution and spatiotemporal change of the supply–demand ratio in the sub-watershed scale.

For spatial management, the grid scale data is not very operable for management, thus we also discuss the supply–demand matching from the sub-watershed scale (Figure 13). The high-value sub-watersheds and low-value sub-watersheds of the supply–demand ratio for FP are both distributed in the midstream area and downstream area. In 2000, the supply–demand ratio of FP in the sub-watershed 35 is the lowest, while in 2015, the supply–demand ratio of FP in the sub-watersheds 35 and 37 are the lowest. From 2000 to 2015, the supply–demand ratios of FP in the sub-watersheds 1, 13, 32, 35, 36, and 37 show a downward trend. For WY, the sub-watersheds with high supply–demand ratio are mainly distributed in the upstream area, and the sub-watersheds with low-value are mainly distributed in the midstream area and downstream area. In 2000 and 2015, the supply–demand ratio of WY in the sub-watersheds 9, 16, 19, and 35 are low. The supply–demand ratio of WY in most sub-watersheds shows a downward trend from 2000 to 2015, and only the supply–demand ratio in the sub-watersheds 2, 6, 7, 8, and 16 increases.

The spatial distribution of the supply–demand ratio of CR and SR is similar in the sub-watershed scale. The supply–demand ratio of SR is lower in the sub-watersheds of midstream area and downstream area, but it is higher in the sub-watersheds of the upstream area. From 2000 to 2015, the supply–demand ratio in the sub-watersheds 3, 10, 24, 26, 35, and 38 increases, while that of other sub-watersheds remains unchanged or decreases. The sub-watersheds with high supply–demand ratio for CR are mainly distributed in the upstream area. In 2000 and 2015, the supply–demand ratio of CR in the sub-watershed 35 is lower. From 2000 to 2015, the supply–demand ratio of CR has no significant change in the sub-watershed 7, and which shows a downward trend in other sub-watersheds.

### 3.4. The Supply–Demand Risks of Four ES

#### 3.4.1. The Supply–Demand Risks in Grid-Scale

**Figure 14:**
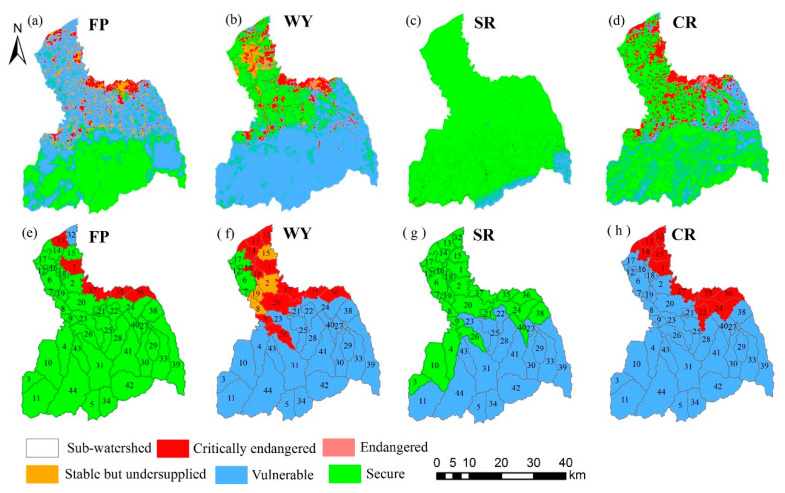
The spatial distribution of supply–demand disk of four ES. The figures (**a**–**d**) are the supply-demand risk of FP, WY, SR, and CR on grid scale, respectively; The figures (**e**–**h**) are the supply-demand risk of FP, WY, SR, and CR on sub-watershed scale, respectively.

According to Figure 14a–d, except for SR, the extremely endangered and endangered areas for FP, WY, and CR are almost the same, and they are mainly located in the midstream area and downstream area. Compared with other ES, there is a “stable but undersupplied” area for WY in the downstream area. There are few areas in the extremely endangered level, endangered level, and stable but undersupplied level for SR, most of which are in a “secure” level.

#### 3.4.2. The Supply–Demand Risks in the Sub-Watershed Scale

Because the distribution of supply–demand risk of ES in the grid scale is relatively scattered, it is not good for regional management. Therefore, this study further discusses the spatial distribution of supply–demand risk from the sub-watershed scale (Figure 14e–h). From the sub-watershed scale, the supply–demand risk level of SR is at a no supply deficit level. However, there are critically endangered levels for the other three ES. The sub-watersheds 1, 13, 35, 36, and 37 are at the extremely endangered level for FP, WY and CR. The sub-watersheds 14, 15, 22, 24, and 32 are at the critically endangered level for CR. The sub-watersheds 9, 14, 16, 18, 20 and 26 are also at the critically endangered level for WY. Meanwhile, the sub-watersheds 2, 8, 15, and 19 are at the “stable but undersupplied” level for WY.

#### 3.4.3. The Supply–Demand Risks at the Overall FRW

From the overall view of the FRW, the current status and change trend of supply–demand ratio and supply change trend of the four ES are not completely consistent (Table 4). Among them, the supply–demand risk level of FP, WY, and CR are the same, all of which are at the “vulnerable” level. The supply–demand risk of SR is at a “secure” level.

### 3.5. Space Management Zoning Based on Supply–Demand Risks of ES

**Figure 15:**
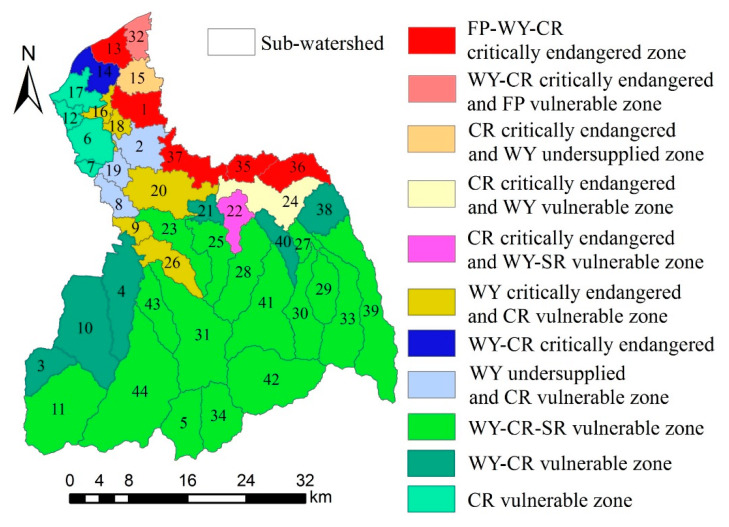
Space management zoning of the FRW.

The FRW is divided into 11 different supply–demand management zones (Figure 15). FP-WY-CR critically endangered zone refers to FP, WY, and CR being all at a critically endangered level and that SR is at a secure level. WY-CR critically endangered and FP vulnerable zone refers to WY and CR being at a critically endangered level, and that FP is at a vulnerable level, while SR is at a secure level. CR critically endangered and WY undersupplied zone refers to CR being at a critically endangered level, and that WY is at a stable but undersupplied level, while FP and SR are at a secure level. CR critically endangered and WY vulnerable zone refers to CR being at a critically endangered level, and that WY is at a vulnerable level, while FP and SR are at a secure level. CR critically endangered and WY vulnerable zone refers to that CR being at a critically endangered level, and that WY and SR are at a vulnerable level, while FP is at a secure level. WY-CR critically endangered zone refers to WY and CR being in a critically endangered level, while FP and SR are at a secure level. WY undersupplied and CR vulnerable zone refers to WY being at a stable but undersupplied level, and that CR is at a vulnerable level, while FP and SR are at a secure level. WY-CR-SR vulnerable zone refers to WY, CR, and SR being at a vulnerable level, while FP is at a secure level. WY-CR vulnerable zone refers to WY and CR being at a vulnerable level, while FP and SR are at a secure level. CR vulnerable zone refers to CR being at a vulnerable level, while the other three ES are at a secure level.

## 4. Discussion

### 4.1. The Factors Influencing Supply–Demand Risks of ES

From the supply side, the weaker ES supply capacity may cause a lower supply–demand ratio of ES, and this may lead to a high level of supply–demand risk (such as critically endangered). For example, the CR capacity of the sub-watershed 35 is relatively weak, which is the cause of insufficient supply of CR in this area. Similar conclusions have been drawn in studies in other regions [54,55]. For example, due to the limited greening trees in urban areas, the carbon storage service of greening trees cannot meet the urban emission demand [54,55]. From the demand side, the higher demand for ES may cause a higher level of supply–demand risk, while the lower demand for ES will cause a lower level of supply–demand risk. For example, in the sub-watersheds 6, 7, 12, and 17, due to the lower demand for WY, the supply–demand risk of WY is in a secure level. While, in the sub-watershed 35, due to the higher demand for WY, the supply–demand risk of WY is in a critically endangered level.

Generally, the supply–demand risk of ES is affected by both supply and demand. The lower supply and the higher demand generally result in higher level of supply–demand risk. For example, in the sub-watershed 35, the land use type is mainly urban land, and the supply capacity of ES is very low. Due to the higher population density, the demand for ES is higher. Therefore, except for SR, the supply–demand risks of the other three ES in the sub-watershed 35 are all critically endangered level. The lower supply and lower demand also cause a lower level of supply–demand risk. For example, the sub-watersheds of upstream area have a low FP capacity. However, due to the steep terrain and sparse population, the demand for ES in these sub-watersheds is also low. Therefore, the supply–demand risk of FP in these sub-watersheds is in a secure level.

### 4.2. The Significance of Supply–Demand Risk Assessment of ES

ES are the prerequisite of human survival and development [11]. How, though, do we determine whether humans face the supply deficit risk of ES? To what extent is the risk? What is the trend? The assessment of these issues can provide a basis for ES management and related policy-making [56]. The supply–demand risk assessment of ES connects the ES supply and demand, and divides the supply–demand risk level of ES from the risk perspective, which provides a new perspective for ES management [34]. Compared with the previous ES risk assessment, the supply–demand risk of ES has two advantages as follows.

(1) Considering the supply and demand of ES, the supply–demand risk of ES combines natural ecosystems and human social systems. The supply–demand risk assessment of ES not only considers the risk of supply degradation of ES, but also introduces the humans’ demand for ES. ES refer to the benefits of human beings from ecosystem, thus the demand for ES should be used as an important reference indicator. Although the supply capacity of ES is poor, and the demand for it is also very low, the supply–demand risk may be relatively low. For example, the sub-watershed 17 in this study has low WY capacity and fresh water demand, and the risk of WY is in a secure level. In this case, there is no need to allocate water resources into this area. However, when the supply capacity of ES is low, and the demand is high, the supply–demand risk level may be higher. For example, in the sub-watershed 14, the WY capacity is lower and the water demand is higher, and the risk level of WY service is critically endangered. In this case, water resources need to be allocated to the sub-watershed 14 in priority. Although the sub-watershed 14 and the sub-watershed 17 are spatially adjacent, the risk levels of supply–demand of WY are quite different. When allocating water resources, they cannot be treated equally, and reasonable allocation of water resources should be implemented according to the threat degree of supply–demand risk.

In the current research, ES management based on the supply–demand matching of ES has been launched. For example, Cui et al. [57] evaluated the supply–demand ratio of ES in three spatial scales of Hulunbuir (local, town, and country), and they analyzed the spatial matching of ES in three scales. They suggested that Hulunbuir should protect natural forest and grassland and give priority to water-saving agriculture. Lorilla et al. [11] calculated the supply–demand ratio of three ES (FP, climate regulation, and recreation) in the Ionian Islands (western Greece), and combined it with hot spot analysis to determine the priority of protected areas. These studies show that the role of the supply–demand matching of ES cannot be ignored in ES management. Therefore, the supply–demand risk assessment of ES can identify the causes of supply–demand risk and provide a reference for the optimal allocation of resources.

(2) The supply–demand risk of ES also considers the change trend of supply–demand ratio of ES and the change trend of ES supply. Compared with static supply–demand matching, this composite and multi-dimensional risk level assessment is more effective for ES management. The supply and demand of ES change dynamically with time, which directly affects the change of supply–demand ratio. The change trend of supply–demand ratio and the change trend of supply will affect the supply–demand risk level of ES. For example, when the supply–demand ratio of the two regions is less than 0, that is, supply deficit, the supply–demand risk in areas where the supply–demand ratio increases is less than the supply–demand risk in areas where the supply–demand ratio decreases. Maron et al. [34] proposed that the trend of supply–demand ratio should be included in the framework of ES supply–demand risk assessment, but the relevant case studies need to be further carried out. Wang et al. [58] evaluated the supply–demand risks of WY in Shaanxi Province based on supply–demand risk framework. They found that the high-risk areas accounted for 13.37% of Shaanxi Province and the low-risk areas accounted for 86.63% in the period 2000–2010. In this study, we also used this supply–demand risk framework to assess the supply–demand risk of ES, and overlay the supply–demand risk areas of four ES assessed. Then, the risk management zoning of the FRW is divided, which can reflect the corresponding ecological environment problems of different regions, and is more accurate for regional ES management.

### 4.3. The Application of Supply–Demand Risk Management Zoning of Multiple ES

The risk management zones based on the division of supply–demand risks of multiple ES can show the risk level of multiple ES. On the one hand, according to the risk level of different ES, the ES with high risk level (critically endangered and endangered) should be controlled and managed preferentially. On the other hand, the risk management zones can also identify the risk level of the same ES in different zones. The identification can guide the spatial allocation of resources between zones through ES flow, to achieve a reasonable allocation of resource [59,60], such as the allocation of FP from low-risk zones (vulnerable and secure) to high-risk zones (critically endangered and endangered).

For the FRW, although the supply–demand risk levels for four ES are low (SR is secure and others is vulnerable), there are also high-level risks for four ES in some local areas. The supply–demand risk levels for four ES are low in the sub-watersheds of upstream area. For example, sub-watersheds 31, 42, and 44 are WY-CR-SR vulnerable zone. Thus, no control measures are required at present. However, because these sub-watersheds are important areas for ecological functions (WY, SR, CR), it is necessary to monitor the change trend of three ES (WY, SR, CR) supply and strictly prevent their degradation. Another example is that the sub-watershed 24 belongs to the CR critically endangered and WY vulnerable zone. In the sub-watershed, CR service should be managed with priority, such as increasing the area of green infrastructure to increase CR supply [61]. Further, FP, WY, and CR are all critically endangered for sub-watersheds 1, 13, 35, 36, and 37. The land use types of these sub-watersheds are mainly urban land, and their population density is large. Therefore, green foundation should be added to these sub-watersheds, and the utilization rate of water resources should be improved. At the same time, these sub-watersheds also need to allocate food and fresh water from other regions to meet the sustainable development of social economy.

In addition, the risk level of the same ES presents different risk levels in different sub-watersheds of the FRW. For example, the supply–demand risk level of WY belongs to vulnerable zones in the sub-watersheds of upstream area(e.g., 31,34,42,44), while it belongs to critically endangered zone in the sub-watersheds of midstream area and downstream area(e.g., 1,13,16,18,20). Therefore, the ES supply–demand risk can be spatially regulated in the form of ES flow.

### 4.4. The Limitations

Due to differences in ES supply and demand assessment methods, there may be differences in the dimensions of supply and demand assessment results. This will affect the calculation of the supply–demand ratio of ES, making it difficult to assess the supply–demand risks of ES. In addition, there are various types of ES. Such as Costanza et al., which divides ES into 17 categories [2], and the Millennium Ecosystem Assessment is divided into 22 categories [11]. If these ES are evaluated, it will take much time and manpower. Therefore, the assessment of regional ES generally selects key regional ES as representatives. Due to the limitations of assessment methods, this study did not fully assess the supply–demand risks of key ES in the FRW (e.g., water quality and air purification not evaluated). In the following research, we will conduct research on other key ES assessment methods. In addition, we will strive to optimize existing evaluation methods.

## 5. Conclusions

The supply–demand risks of four ES (FP, WY, SR, CR) are assessed and risk management zones are divided, which is of great significance for the sustainable management of ES. For the whole FRW, the supply–demand risks of FP, WY and CR are vulnerable, while the supply–demand risk of SR is secure. In terms of sub-watershed, except for the absence of high-risk areas for SR, there are high-risk areas for the other three ES. These high-risk areas are mainly distributed in the midstream area and downstream area of the FRW. The FRW is divided into 11 supply–demand risk management zones, such as FP-WY-CR critically endangered zone, WY-CR critically endangered and FS vulnerable zone, CR critically endangered and WY undersupplied zone. The supply–demand risk management zones can identify the supply–demand risk status of different ES in different zones, and provide basis for the rational allocation of regional resources, to facilitate the sustainable management of regional ES.

## Figures and Tables

**Table 1:** The data sources mapped by the supply and demand of ecosystem services (ES).

Data Types	Data Formats	Sources and Descriptions
Land use	Shapefile, grid, 30 m resolution	National Ecosystem Survey and Assessment of China (2000–2010) Remote Sensing Survey and Assessment Project and Investigation, Assessment Project of National Ecological Situation Change project (2010–2015). The primary classification accuracy is about 97%, and the secondary classification accuracy is about 86%.
Digital elevation model (DEM)	Grid, 30 m resolution	The data came from the Geospatial data cloud. (http://www.gscloud.cn/)
Precipitation, potential evapotranspiration	Grid, 30 m resolution	The precipitation data came from the “Hydrological Yearbook of the People’s Republic of China”—“Hydrological Data of the Yellow River Basin”, Volume 3 (2000–2015) and the China Meteorological Data Network; potential evapotranspiration data came from the China Meteorological Data Network, (http://data.cma.cn/)
Soil properties	Shapefile	The data came from “Shaanxi soil [36] and “The second survey of soil (http://vdb3.soil.csdb.cn/extend/jsp/introduction)
Food production, Energy consumption	Excel, text	The data came from the “Chang’an Statistical Yearbook” (2001, 2016) [37,38] and “Xi’an Statistical Yearbook” (2001, 2016) [39,40] (http://tjj.xa.gov.cn/)
Fresh water consumption	Excel, text	Xi’an water resources bulletin (2015) (http://swj.xa.gov.cn/)and Shaanxi water resources bulletin (2000, 2015) (http://slt.shaanxi.gov.cn/)
Population	Excel	The fifth and sixth census of China, 1% population survey of Shaanxi Province in 2015 (http://www.shaanxi.gov.cn/info/iList.jsp?tm_id=166&cat_id=10003&info_id=452 )
Normalized difference vegetation index (NDVI)	Grid, 50 m resolution	National Ecosystem Survey and Assessment of China (2000–2010) Remote Sensing Survey and Assessment Project and Investigation, Assessment Project of National Ecological Situation Change project (2010–2015).
Nighttime lights	Grid, 250 m resolution	The night lights from DMSP/OLS in 2000 (http://www.resdc.cn/data.aspx?DATAID=213) and from Suomi-NPP in 2015 (https://www.ngdc.noaa.gov/eog/download.html)
Food calories per capita	Text	“China food and Nutrition Development Program (2001–2010)” (http://www.gov.cn/zhengce/content/201610/11/content_5117329.htm) and “China food and Nutrition Development Program (2014–2020)” (http://www.gov.cn/zhengce/content/2014-02/10/content_8638.htm)

Note: The data with web addresses can be obtained from open source.

**Table 2:** The classification of supply–demand risks of ES.

Current Situation of Supply–Demand Ratio	Trend of Supply–Demand Ratio	Trend of the Supply	Level of Supply–Demand Risk
R < 0	R_tr_ < 0		critically endangered
R < 0	R_tr_ ≥ 0	S_tr_ < 0	endangered
R < 0	R_tr_ ≥ 0	S_tr_ > 0	stable but undersupplied
R ≥ 0	R_tr_ < 0		vulnerable
R ≥ 0	R_tr_ ≥ 0		secure

Note: R is the supply–demand ratio; R_tr_ represents the change trend in the supply–demand ratio in a certain period, less than 0 is a decline, greater than 0 is an increase; S_tr_ is the change trend of ES supply in a certain period, less than 0 is a decline, greater than 0 is an increase.

**Table 3:** The mean of supply–demand ratio for four ES in the FRW.

Year	FS	WY	SR	CR
2000	0.067	0.054	0.061	0.374
2015	0.028	0.024	0.063	0.059

**Table 4:** The status and trends of supply–demand ratio, and change trend of ES supply for four ES.

Reference Indicator	FP	WY	SR	CR
*R*	>0	>0	>0	>0
*R_tr_*	<0	<0	>0	<0
*S_tr_*	>0	>0	>0	<0

## Appendix A

**Table A1:** The supply amount and changes of four ES from 2000 to 2015.

ES	2000	2015	Change Rate
FP	2.16 × 10^12^ kcal	2.41 × 10^12^ kcal	+11.59%
WY	5.86 × 10^9^ m^3^	5.93 × 10^9^ m^3^	+1.2%
SR	1.33 × 10^8^ t	2.07 × 10^8^ t	+55%
CR	1.32 × 10^7^ t	1.25 × 10^7^ t	−5.15%

**Table A2:** The demand amount and changes of four ES from 2000 to 2015.

ES	2000	2015	Change Rate
FP	7.45 × 10^11^ kcal	10.38 × 10^11^ kcal	+39.37%
WY	2.45 × 10^8^ m^3^	3.77 × 10^8^ m^3^	+53.88%
SR	4.38 × 10^6^ t	5.97 × 10^6^ t	+36.3%
CR	9.93 × 10^5^ t	3.13 × 10^6^ t	+215.51%
